# (*E*)-*N*-Ethyl-2-[(*E*)-3-(hy­droxy­imino)­butan-2-yl­idene]hydrazinecarbothio­amide

**DOI:** 10.1107/S1600536812028632

**Published:** 2012-07-25

**Authors:** Halema Shaban Abduelftah, Amna Qasem Ali, Naser Eltaher Eltayeb, Siang Guan Teoh, Hoong-Kun Fun

**Affiliations:** a School of Chemical Sciences, Universiti Sains Malaysia, Penang, Malaysia; b University of Sabha, Libya; c School of Chemical Sciences, Universiti Sains Malaysia, Minden, Penang, Malaysia; d Faculty of Science, Sabha University, Libya; e Department of Chemistry, International University of Africa, Khartoum, Sudan; f X-ray Crystallography Unit, School of Physics,Universiti Sains Malaysia, 11800 USM, Penang, Malaysia

## Abstract

In the crystal structure of the title compound, C_7_H_14_N_4_OS, mol­ecules are linked through N—H⋯S and O—H⋯N hydrogen bonds and C—H⋯S interactions, forming chains propagating along [21-1].

## Related literature

For related structures, see:Abduelftah *et al.* (2012*a* ▶,*b* ▶); Choi *et al.* (2008 ▶). For the biological activity and pharmacological properties of thio­semicarbazones and their metal complexes, see: Cowley *et al.* (2002 ▶); Ming (2003 ▶). For graph-set analysis of hydrogen bonds, see: Bernstein *et al.* (1995 ▶).
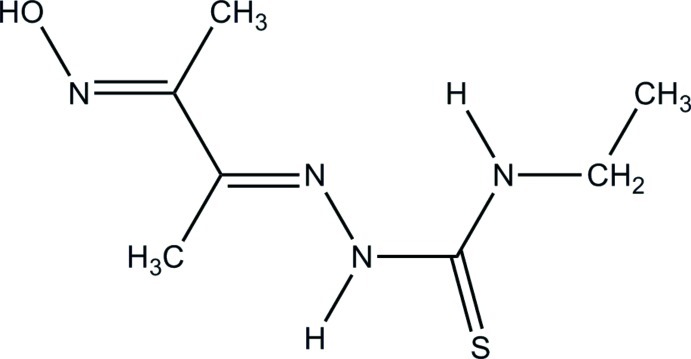


## Experimental

### 

#### Crystal data

C_7_H_14_N_4_OS
*M* *_r_* = 202.28
Triclinic, 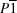

*a* = 5.7065 (2) Å
*b* = 9.0632 (3) Å
*c* = 10.7109 (4) Å
α = 71.309 (1)°
β = 76.318 (1)°
γ = 86.420 (1)°
*V* = 509.80 (3) Å^3^
*Z* = 2
Mo *K*α radiation
μ = 0.29 mm^−1^
*T* = 100 K
0.57 × 0.20 × 0.07 mm

#### Data collection

Bruker APEXII CCD diffractometer
Absorption correction: multi-scan (*SADABS*; Bruker, 2005 ▶) *T*_min_ = 0.854, *T*_max_ = 0.979
15442 measured reflections
4093 independent reflections
3648 reflections with *I* > 2σ(*I*)
*R*_int_ = 0.023

#### Refinement

*R*[*F* ^2^ > 2σ(*F* ^2^)] = 0.032
*wR*(*F* ^2^) = 0.093
*S* = 1.08
4093 reflections
121 parameters
H-atom parameters constrained
Δρ_max_ = 0.46 e Å^−3^
Δρ_min_ = −0.33 e Å^−3^

### 

Data collection: *APEX2* (Bruker, 2005 ▶); cell refinement: *SAINT* (Bruker, 2005 ▶); data reduction: *SAINT*; program(s) used to solve structure: *SHELXTL* (Sheldrick, 2008 ▶); program(s) used to refine structure: *SHELXTL*; molecular graphics: *SHELXTL*; software used to prepare material for publication: *SHELXTL* and *PLATON* (Spek, 2009 ▶).

## Supplementary Material

Crystal structure: contains datablock(s) I, global. DOI: 10.1107/S1600536812028632/ng5276sup1.cif

Structure factors: contains datablock(s) I. DOI: 10.1107/S1600536812028632/ng5276Isup2.hkl

Supplementary material file. DOI: 10.1107/S1600536812028632/ng5276Isup3.cml

Additional supplementary materials:  crystallographic information; 3D view; checkCIF report

## Figures and Tables

**Table 1:** Hydrogen-bond geometry (Å, °)

*D*—H⋯*A*	*D*—H	H⋯*A*	*D*⋯*A*	*D*—H⋯*A*
O1—H1*O*1⋯N1^i^	0.85	2.00	2.7876 (10)	154
N3—H1*N*3⋯S1^ii^	0.87	2.75	3.6124 (8)	171
C4—H4*A*⋯S1^ii^	0.98	2.64	3.4302 (12)	138

Symmetry codes: (i) 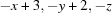; (ii) 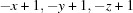.

**Fig. 1.**
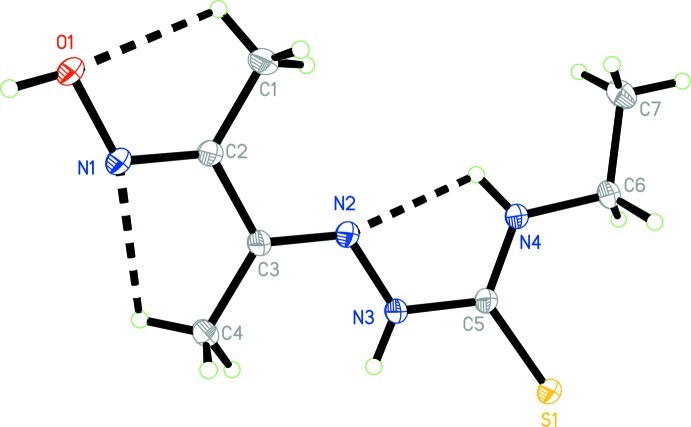
The molecular structure of the title compound, with 50% probability displacement ellipsoids and the atom-numbering scheme.

**Fig. 2.**
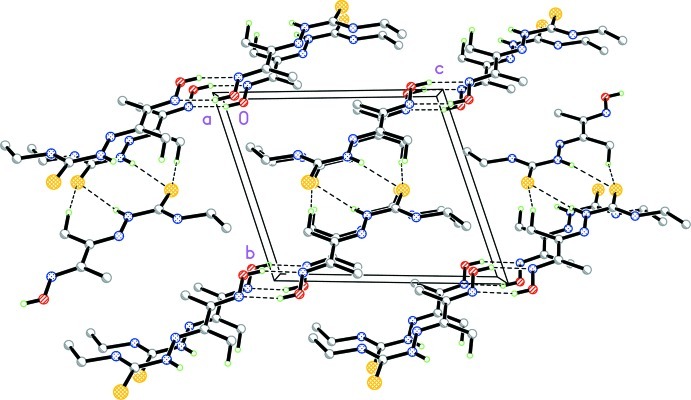
The crystal packing of the title compound viewed down the a axis. Hydrogen bonds are shown as dashed lines.

## supplementary crystallographic information

### Comment

Thiosemicarbazones and their metal complexes have attracted significant attention because of their wide-ranging biological and pharmacological activities related to specific structures as well as chemical properties (Cowley *et al.*, 2002; Ming, 2003). In this paper we report the crystal structure of the title compound (Fig. 1).

In the title compound, C_7_H_14_N_4_OS, the butyl chain is the longest carbon-carbon chain with the hydroxylamine group bound to C2 and the *N*-ethylhydrazinecarbothioamide moiety bound to C3.

Cyclic intramolecular N4—H1N4···N2, C1—H1A···O1 and C4—H4B···N1 hydrogen-bonding interactions [graph set S(5), (Bernstein *et al.*, 1995)] are present (Table 1). In the crystal molecules are connected through intermolecular O1—H1O1···N1, N3—H1N3···S1 and C4—H4A···S1 hydrogen bonds into infinite chains which propagate along [2 1 - 1] (Table 1, Fig.2).

### Experimental

The ligand was prepared by mixing a solution of 2,3-butanedione monoxime (1.01 g, 1 mmol) in EtOH (20 ml) with a solution of 4-ethyl-3-thiosemicarbzide (1.19 g, 1 mmol) in EtOH (20 ml). On adding a few drops of glacial acetic acid to the mixture, a solution of yellowish-white color was formed. The reaction mixture then was heated under reflux with stirring for 3 hrs. The mixture was filtered and left to cool; a white precipitate was formed, then collected by filtration and washed by cold EtOH. Colorless crystal was grown by slow evaporation of EtOH at room temperature, yield (66%).

### Refinement

The H atoms were positioned geometrically and refined using a riding model with O—H = 0.85; *U*_iso_(H) = 1.5Ueq(O), N—H = 0.87; *U*_iso_(H) = 1.2Ueq(N), C—H = 0.98; *U*_iso_(H) = 1.5Ueq(C) for methyl groups and C—H = 0.99; *U*_iso_(H) = 1.2Ueq(C) for methylene group. The highest residual electron density peak is located 0.64 Å from C2 and the deepest hole is located 0.16 Å from H4B.

### Crystal data

**Table:**

C_7_H_14_N_4_OS	*Z* = 2
*M**_r_* = 202.28	*F*(000) = 216
Triclinic, *P*1	*D*_x_ = 1.318 Mg m^−^^3^
Hall symbol: -P 1	Mo *K*α radiation, λ = 0.71073 Å
*a* = 5.7065 (2) Å	Cell parameters from 8235 reflections
*b* = 9.0632 (3) Å	θ = 3.6–35.1°
*c* = 10.7109 (4) Å	µ = 0.29 mm^−^^1^
α = 71.309 (1)°	*T* = 100 K
β = 76.318 (1)°	Plate, colourless
γ = 86.420 (1)°	0.57 × 0.20 × 0.07 mm
*V* = 509.80 (3) Å^3^	

### Data collection

**Table:**

Bruker APEXII CCD diffractometer	4093 independent reflections
Radiation source: fine-focus sealed tube	3648 reflections with *I* > 2σ(*I*)
Graphite monochromator	*R*_int_ = 0.023
φ and ω scans	θ_max_ = 34.0°, θ_min_ = 2.6°
Absorption correction: multi-scan (*SADABS*; Bruker, 2005)	*h* = −8→8
*T*_min_ = 0.854, *T*_max_ = 0.979	*k* = −14→14
15442 measured reflections	*l* = −16→16

### Refinement

**Table:**

Refinement on *F*^2^	Primary atom site location: structure-invariant direct methods
Least-squares matrix: full	Secondary atom site location: difference Fourier map
*R*[*F*^2^ > 2σ(*F*^2^)] = 0.032	Hydrogen site location: inferred from neighbouring sites
*wR*(*F*^2^) = 0.093	H-atom parameters constrained
*S* = 1.08	*w* = 1/[σ^2^(*F*_o_^2^) + (0.0432*P*)^2^ + 0.1608*P*] where *P* = (*F*_o_^2^ + 2*F*_c_^2^)/3
4093 reflections	(Δ/σ)_max_ = 0.001
121 parameters	Δρ_max_ = 0.46 e Å^−^^3^
0 restraints	Δρ_min_ = −0.33 e Å^−^^3^

### Special details

**Table:**

Geometry. All e.s.d.'s (except the e.s.d. in the dihedral angle between two l.s. planes) are estimated using the full covariance matrix. The cell e.s.d.'s are taken into account individually in the estimation of e.s.d.'s in distances, angles and torsion angles; correlations between e.s.d.'s in cell parameters are only used when they are defined by crystal symmetry. An approximate (isotropic) treatment of cell e.s.d.'s is used for estimating e.s.d.'s involving l.s. planes.
Refinement. Refinement of *F*^2^ against ALL reflections. The weighted *R*-factor *wR* and goodness of fit *S* are based on *F*^2^, conventional *R*-factors *R* are based on *F*, with *F* set to zero for negative *F*^2^. The threshold expression of *F*^2^ > σ(*F*^2^) is used only for calculating *R*-factors(gt) *etc*. and is not relevant to the choice of reflections for refinement. *R*-factors based on *F*^2^ are statistically about twice as large as those based on *F*, and *R*- factors based on ALL data will be even larger.

### Fractional atomic coordinates and isotropic or equivalent isotropic displacement parameters (Å^2^)

**Table:**

	*x*	*y*	*z*	*U*_iso_*/*U*_eq_	
S1	0.44242 (4)	0.52051 (3)	0.69791 (2)	0.02043 (7)	
O1	1.61220 (13)	1.04092 (8)	0.11132 (7)	0.02309 (14)	
H1O1	1.6452	1.0633	0.0256	0.035*	
N1	1.41316 (13)	0.94009 (9)	0.15010 (7)	0.01695 (13)	
N2	1.01528 (13)	0.74755 (9)	0.45088 (7)	0.01587 (13)	
N3	0.81401 (13)	0.65358 (9)	0.50040 (7)	0.01734 (13)	
H1N3	0.7419	0.6218	0.4504	0.021*	
N4	0.82678 (13)	0.66298 (9)	0.71029 (7)	0.01754 (13)	
H1N4	0.9652	0.7085	0.6662	0.021*	
C1	1.41628 (18)	0.94145 (12)	0.37977 (9)	0.02285 (17)	
H1A	1.5830	0.9797	0.3405	0.034*	
H1B	1.4111	0.8518	0.4613	0.034*	
H1C	1.3154	1.0244	0.4035	0.034*	
C2	1.32385 (15)	0.89332 (10)	0.27889 (8)	0.01613 (14)	
C3	1.11201 (15)	0.78830 (10)	0.32272 (8)	0.01645 (14)	
C4	1.02391 (19)	0.73958 (13)	0.22066 (9)	0.0260 (2)	
H4A	0.9612	0.6324	0.2616	0.039*	
H4B	1.1576	0.7453	0.1425	0.039*	
H4C	0.8953	0.8091	0.1911	0.039*	
C5	0.70823 (14)	0.61747 (10)	0.63536 (8)	0.01525 (13)	
C6	0.73697 (16)	0.63713 (11)	0.85512 (8)	0.01993 (15)	
H6A	0.7165	0.5240	0.9036	0.024*	
H6B	0.5777	0.6864	0.8714	0.024*	
C7	0.91280 (18)	0.70594 (12)	0.90870 (10)	0.02417 (18)	
H7A	0.8508	0.6882	1.0059	0.036*	
H7B	0.9315	0.8181	0.8612	0.036*	
H7C	1.0696	0.6559	0.8936	0.036*	

### Atomic displacement parameters (Å^2^)

**Table:**

	*U*^11^	*U*^22^	*U*^33^	*U*^12^	*U*^13^	*U*^23^
S1	0.01501 (10)	0.03141 (12)	0.01420 (10)	−0.00775 (7)	0.00028 (6)	−0.00740 (8)
O1	0.0233 (3)	0.0273 (3)	0.0159 (3)	−0.0142 (2)	−0.0004 (2)	−0.0031 (2)
N1	0.0172 (3)	0.0177 (3)	0.0139 (3)	−0.0061 (2)	−0.0013 (2)	−0.0027 (2)
N2	0.0154 (3)	0.0189 (3)	0.0117 (3)	−0.0038 (2)	−0.0016 (2)	−0.0030 (2)
N3	0.0163 (3)	0.0242 (3)	0.0109 (3)	−0.0066 (2)	−0.0010 (2)	−0.0050 (2)
N4	0.0158 (3)	0.0247 (3)	0.0119 (3)	−0.0052 (2)	−0.0012 (2)	−0.0060 (2)
C1	0.0260 (4)	0.0279 (4)	0.0160 (4)	−0.0094 (3)	−0.0062 (3)	−0.0060 (3)
C2	0.0174 (3)	0.0173 (3)	0.0129 (3)	−0.0041 (2)	−0.0032 (2)	−0.0032 (3)
C3	0.0175 (3)	0.0197 (3)	0.0116 (3)	−0.0050 (3)	−0.0021 (2)	−0.0040 (3)
C4	0.0287 (4)	0.0360 (5)	0.0138 (3)	−0.0161 (4)	−0.0015 (3)	−0.0081 (3)
C5	0.0140 (3)	0.0191 (3)	0.0117 (3)	−0.0020 (2)	−0.0017 (2)	−0.0040 (3)
C6	0.0202 (3)	0.0278 (4)	0.0118 (3)	−0.0039 (3)	−0.0012 (3)	−0.0073 (3)
C7	0.0253 (4)	0.0323 (5)	0.0194 (4)	−0.0007 (3)	−0.0078 (3)	−0.0122 (3)

### Geometric parameters (Å, º)

**Table:**

S1—C5	1.6823 (8)	C1—H1B	0.9800
O1—N1	1.4004 (9)	C1—H1C	0.9800
O1—H1O1	0.8499	C2—C3	1.4753 (11)
N1—C2	1.2891 (10)	C3—C4	1.4966 (12)
N2—C3	1.2913 (10)	C4—H4A	0.9800
N2—N3	1.3676 (10)	C4—H4B	0.9800
N3—C5	1.3674 (10)	C4—H4C	0.9800
N3—H1N3	0.8699	C6—C7	1.5182 (13)
N4—C5	1.3326 (10)	C6—H6A	0.9900
N4—C6	1.4594 (11)	C6—H6B	0.9900
N4—H1N4	0.8699	C7—H7A	0.9800
C1—C2	1.4955 (12)	C7—H7B	0.9800
C1—H1A	0.9800	C7—H7C	0.9800
			
N1—O1—H1O1	101.9	C3—C4—H4A	109.5
C2—N1—O1	113.41 (7)	C3—C4—H4B	109.5
C3—N2—N3	118.88 (7)	H4A—C4—H4B	109.5
C5—N3—N2	117.92 (7)	C3—C4—H4C	109.5
C5—N3—H1N3	117.7	H4A—C4—H4C	109.5
N2—N3—H1N3	124.1	H4B—C4—H4C	109.5
C5—N4—C6	123.53 (7)	N4—C5—N3	116.43 (7)
C5—N4—H1N4	114.6	N4—C5—S1	123.74 (6)
C6—N4—H1N4	121.9	N3—C5—S1	119.83 (6)
C2—C1—H1A	109.5	N4—C6—C7	110.08 (7)
C2—C1—H1B	109.5	N4—C6—H6A	109.6
H1A—C1—H1B	109.5	C7—C6—H6A	109.6
C2—C1—H1C	109.5	N4—C6—H6B	109.6
H1A—C1—H1C	109.5	C7—C6—H6B	109.6
H1B—C1—H1C	109.5	H6A—C6—H6B	108.2
N1—C2—C3	114.67 (7)	C6—C7—H7A	109.5
N1—C2—C1	124.68 (7)	C6—C7—H7B	109.5
C3—C2—C1	120.63 (7)	H7A—C7—H7B	109.5
N2—C3—C2	114.69 (7)	C6—C7—H7C	109.5
N2—C3—C4	125.37 (8)	H7A—C7—H7C	109.5
C2—C3—C4	119.93 (7)	H7B—C7—H7C	109.5
			
C3—N2—N3—C5	−177.16 (8)	N1—C2—C3—C4	2.37 (13)
O1—N1—C2—C3	179.32 (7)	C1—C2—C3—C4	−179.18 (9)
O1—N1—C2—C1	0.93 (13)	C6—N4—C5—N3	178.54 (8)
N3—N2—C3—C2	178.30 (7)	C6—N4—C5—S1	−1.47 (13)
N3—N2—C3—C4	−0.76 (14)	N2—N3—C5—N4	−7.33 (12)
N1—C2—C3—N2	−176.75 (8)	N2—N3—C5—S1	172.68 (6)
C1—C2—C3—N2	1.71 (12)	C5—N4—C6—C7	−178.86 (8)

### Hydrogen-bond geometry (Å, º)

**Table:**

*D*—H···*A*	*D*—H	H···*A*	*D*···*A*	*D*—H···*A*
O1—H1*O*1···N1^i^	0.85	2.00	2.7876 (10)	154
N3—H1*N*3···S1^ii^	0.87	2.75	3.6124 (8)	171
N4—H1*N*4···N2	0.87	2.17	2.6055 (10)	111
C1—H1*A*···O1	0.98	2.30	2.6970 (11)	103
C4—H4*A*···S1^ii^	0.98	2.64	3.4302 (12)	138
C4—H4*B*···N1	0.98	2.39	2.7636 (14)	102

Symmetry codes: (i) −*x*+3, −*y*+2, −*z*; (ii) −*x*+1, −*y*+1, −*z*+1.
